# Safety and effectiveness of lenalidomide in Japanese patients with relapsed/refractory ATLL: post-marketing surveillance

**DOI:** 10.1007/s12185-024-03864-3

**Published:** 2024-11-21

**Authors:** Tohru Miyazaki, Shuji Uno, Hiroaki Fujimori, Yoko Motegi

**Affiliations:** 1 Hematology, Japan Medical, Bristol Myers Squibb, Otemachi One Tower, 1-2-1 Otemachi, Chiyoda-Ku, Tokyo, 100-0004 Japan; 2 PMS Operations, Worldwide Patient Safety Japan, Bristol Myers Squibb, Tokyo, Japan

**Keywords:** Adult T-cell leukemia/lymphoma, Japanese patients, Lenalidomide, Post-marketing surveillance, Relapsed/refractory

## Abstract

Lenalidomide is an oral immunomodulatory agent approved for the treatment of relapsed/refractory adult T-cell leukemia/lymphoma (ATLL) in Japan. Post-marketing surveillance (PMS) was conducted to confirm its safety and effectiveness. From April 2017 until April 2020, safety data were obtained for 77 patients and effectiveness data for 65 patients (31.2% of patients had progressive disease as the best response to their most recent prior regimen). Forty-nine patients (63.6%) in the safety analysis set experienced an adverse drug reaction (ADR). Grade ≥ 3 ADRs occurred in 42.9%. The most common Grade ≥ 3 ADRs were neutrophil count decreased/neutropenia and platelet count decreased/thrombocytopenia (11.7% each). Serious ADRs occurred in 26 patients. Five patients had previously received allogeneic hematopoietic stem cell transplantation. Among these, one experienced acute graft versus host disease (GvHD) during lenalidomide administration and two responded to lenalidomide. Effectiveness analysis showed that an objective response was achieved in 29.2% of patients. No statistically significant differences were observed in the objective response rates of patients aged < 70 versus those aged ≥ 70 years (33.3% vs 28.0%, respectively; *p* = 0.6904). No new safety signals were observed in this PMS, and lenalidomide demonstrated a favorable benefit-risk balance in Japanese patients with ATLL.

**Supplementary Information:**

The online version contains supplementary material available at 10.1007/s12185-024-03864-3.

## Introduction

Adult T-cell leukemia/lymphoma (ATLL) is a rare and aggressive peripheral T-cell lymphoma caused by the human T-cell leukemia virus type I (HTLV-1) [1]. HTLV-1 infection is endemic in Japan [2], with nationwide estimates of 0.65 million HTLV-1 carriers in 2020–2021 based on the screening results of blood donors [3] and an estimated annual incidence rate of 3.8 newly infected individuals per 100,000 person-years in 2016 [4].

A 2016–2020 hospital-based multicenter retrospective study of 984 Japanese patients with ATLL (median age at diagnosis: 69 years) found acute-type ATLL to be the most common ATLL subtype (51.9%), followed by lymphoma-type ATLL (24.9%), chronic-type ATLL (12.5%), and smoldering-type ATLL (10.7%) [5].

Individuals with ATLL typically have poor disease prognoses and often develop resistance to intensive first-line chemotherapy regimens [6–8]. In patients with aggressive forms of ATLL, allogeneic hematopoietic stem cell transplantation (allo-HSCT) is considered the sole, potentially curative, therapeutic option (provided the patient meets transplant eligibility criteria) [9]. However, as almost half of patients diagnosed with ATLL are aged ≥ 70 years, many of whom are frail with multiple comorbidities, dose-intensified cytotoxic chemotherapy followed by allo-HSCT is rarely a viable treatment option in these populations [10].

Lenalidomide is an oral immunomodulatory agent that specifically targets cereblon, an E3 ubiquitin ligase that mediates the degradation of various regulatory proteins involved in major signal transduction cascades underlying development and tissue homeostasis [11]. When bound to lenalidomide, cereblon increases the degradation of specific signaling regulators (e.g., Ikaros and Aiolos), thereby inhibiting cancerous growth [12, 13].

Preclinical studies of lenalidomide in B-cell malignancies reported that lenalidomide enhanced expansion of natural killer T cells, increased antibody-dependent cellular cytotoxicity in rituximab-treated non-Hodgkin’s lymphoma cell lines, reduced proliferation of malignant cells, and restored bone marrow function in other cancer cell lines [14–16]. Based on these results, lenalidomide was also investigated in T-cell malignancies.

In a single-arm, phase 2 study (ATLL-002) of patients with relapsed/recurrent ATLL who had received at least one prior anti-ATLL chemotherapy, oral lenalidomide (25 mg/day) displayed clinically meaningful antitumor activity and an acceptable safety profile [17]. Based on the results of ATLL-002, oral lenalidomide monotherapy was approved in Japan as an orphan drug for treating relapsed/refractory (R/R) ATLL [18]. However, as the ATLL-002 study population was limited to 26 patients and excluded refractory patients, along with the relatively long time since the last treatment for ATLL (median 234.5 days) and the limited observation period (median 3.9 months), further assessment of the safety and effectiveness of lenalidomide in a real-world setting was warranted.

This post-marketing surveillance (PMS) assessed the safety and effectiveness of lenalidomide in patients with R/R ATLL treated in routine clinical practice in Japan.

## Materials and methods

### Study design

This was a prospective PMS of patients with R/R ATLL who were treated with lenalidomide in Japan. The primary objectives were to evaluate the incidence of adverse drug reactions (ADRs) occurring during lenalidomide treatment, assess the effectiveness of lenalidomide based on response rates, and identify factors that may influence the effectiveness/safety of lenalidomide in Japanese patients with R/R ATLL in a real-world setting.

In accordance with the Japanese Pharmaceutical and Medical Devices Agency Act, this PMS was conducted in compliance with the regulatory requirements stipulated in the Good Post-Marketing Study Practice guidelines of Japan, and the protocol was approved by the Ministry of Health, Labour and Welfare prior to initiation. Approval from institutional review boards from each participating site and written informed consent from patients were not mandated according to the ministerial ordinance. If individual medical institutions required ethical approval and consent, these were obtained.

The planned enrolment and survey periods were 3 and 4.5 years, respectively, from 1 month after the initial lenalidomide approval. Patients were enrolled in the PMS by prescribing physicians within 14 days of lenalidomide initiation. Prescribing physicians completed case report forms (CRFs) throughout the observation period (6 months from the date of lenalidomide initiation). CRFs were completed for two separate periods; CRF1 recorded patient data from baseline until 2 months after initiation, and CRF2 collected data from months 3–6 after initiation. The CRFs collected data on baseline demographics and clinical history as well as information on past complications, lenalidomide treatment (dose frequency, daily dose, first/last day of treatment, and reasons for any dose changes/discontinuation), confirmation of survival status, evaluation of treatment effect, presence of ADRs and associated prophylactic treatments.

### PMS participants and treatment

Patients diagnosed with R/R ATLL receiving lenalidomide after April 3, 2017 (PMS start date) at medical institutions contracted for this PMS were enrolled into a central registry. Patients received 25 mg oral lenalidomide capsules, once daily and were observed for up to 6 months or until treatment was discontinued. The dose could be adjusted according to the patient's condition.

### Definition of analysis sets

Patients were excluded from the safety (SAS) and effectiveness analysis sets (EAS) if they: had previously been treated with lenalidomide; did not have a CRF signed off by the contracted physician; were from an uncontracted institution; or for whom the presence/absence of ADRs could not be determined. In addition to the exclusion criteria above, the EAS also excluded patients who used any antitumor drugs other than lenalidomide during the PMS period; or had undetermined antitumor effectiveness results.

**Fig. 1:**
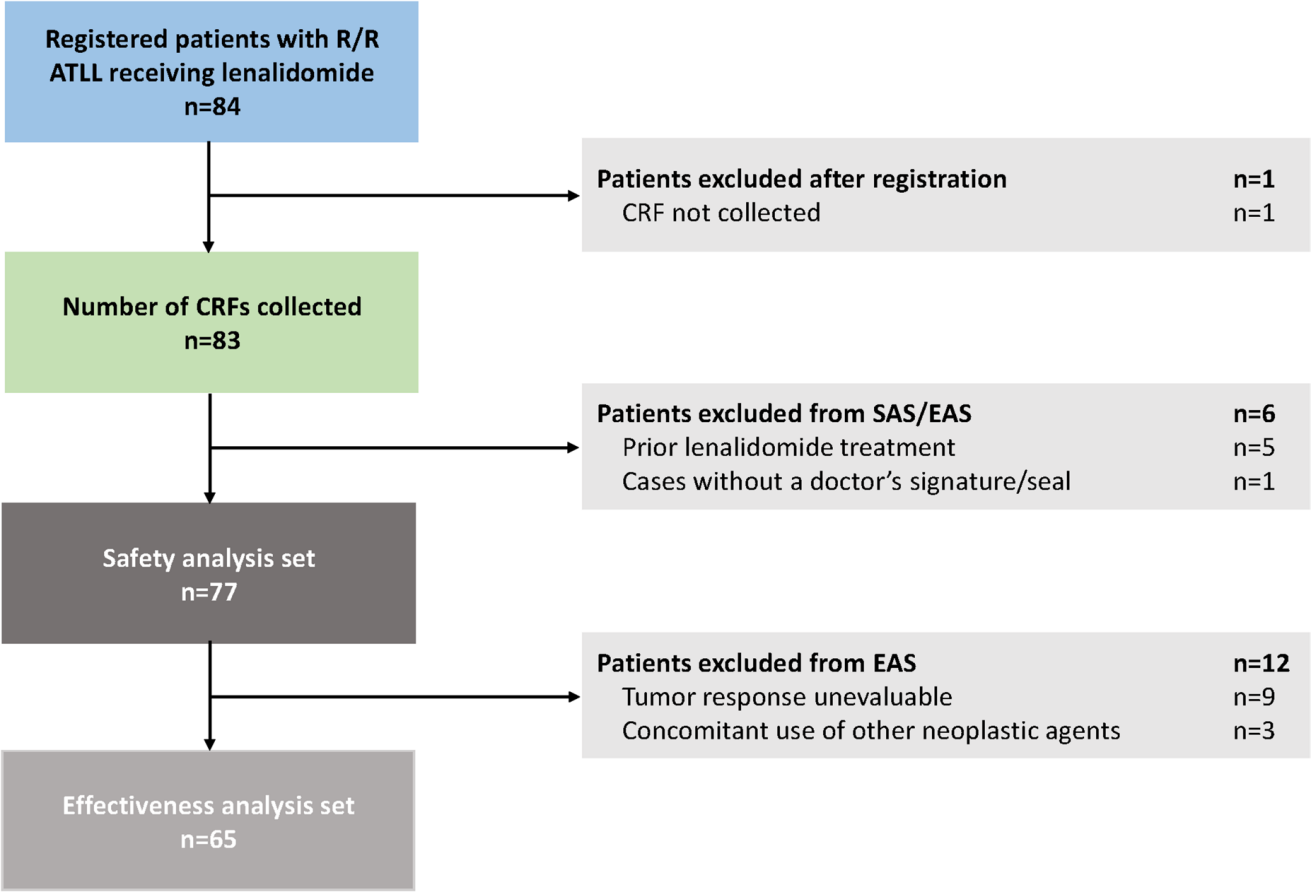
Patient disposition. *ATLL* adult T-cell leukemia/lymphoma, *CRF* case report form, *EAS* effectiveness analysis set, *R/R* relapsed/refractory, *SAS* safety analysis set

For patients who discontinued lenalidomide before the end of the observation period, safety and effectiveness data within 28 days of the last lenalidomide dose were included (Fig. 1).

### Assessments

#### Safety

Prescribing physicians assessed the causality and severity of each ADR. ADRs were classified according to the system organ class (SOC) and preferred term (PT) from the Japanese version of the International Council for Harmonisation of Technical Requirements for Pharmaceuticals for Human Use (ICH) International Glossary of Medical Terms (MedDRA/J) version 25.1 [19]. ADRs were defined as any adverse events (AEs) for which a relationship to lenalidomide could not be ruled out (including unknown events). Serious ADRs (SADRs) were any events that resulted in death, were life-threatening, resulted in permanent or significant disability, or required hospitalization or an extension of a hospital stay.

#### Effectiveness

The prescribing physician assessed effectiveness according to OS and best overall response, classified as either complete response (CR), partial response (PR), stable disease (SD), or progressive disease (PD) according to the Japanese Clinical Oncology Group (JCOG) version of the Criteria for Determining Treatment Efficacy for ATLL [20]. Definitions of the response criteria are shown in Supplementary Table S1.

### Statistical analysis

The target sample size was 80 patients. Assuming that ADRs occur at a similar frequency in routine clinical practice as reported in clinical trials, and based on the ATLL-002 study results (where the lowest incidence of AEs was 3.8%) [17], approximately 80 patients were required to collect safety data with 95% power.

Data were summarized using descriptive statistics, including means, medians, 95% confidence intervals (CIs) and standard deviation (sd) for continuous variables, and the number and percentage of patients for categorical variables. Missing data were labelled as unknown. Statistical significance for background factors which may be influencing lenalidomide response rates in the EAS were calculated using the Chi-squared test for pair-wise comparisons or the Cochran-Armitage test for background factors with three or more categories. Unknown data were not included in the statistical testing. The significance level was set at 5.0% on both sides.

All statistical analyses were conducted by the contract research organization EPS Co., Ltd (Tokyo, Japan) using Statistical Analysis System software, version 9.4 or later (SAS Institute, Inc., Cary, NC, USA).

## Results

### Patient population

From April 3, 2017 to April 2, 2020, 84 patients from 50 medical institutions were enrolled; 83 patients met all screening and inclusion criteria and were included in the PMS. The SAS and EAS included 77 and 65 patients, respectively (Fig. 1).

Six patients were excluded from the SAS due to previous treatment with lenalidomide (*n* = 5) and no physician signature on the CRF (*n* = 1). A further 12 patients were excluded from the EAS due to unevaluable antitumor responses (*n* = 9) or concurrent use of other antineoplastic drugs (*n* = 3).

Forty-seven patients (61.0%) in the SAS were male and the majority (77.9%) were aged ≥ 70 years; 22.1% were aged < 70 years (Table 1). The most common ATLL subtype at baseline was acute-type (44.2%), followed by lymphoma-type (40.3%) and chronic-type ATLL with poor prognostic factors (i.e., high lactate dehydrogenase, high blood urea nitrogen, and low albumin levels; 10.4%) [21]. According to the Ann Arbor classification, a greater proportion of patients had stage IV disease (59.7%) at onset than stage I, II, or III (9.1%, 3.9%, and 23.4%, respectively). The Eastern Cooperative Oncology Group Performance Status (ECOG PS) score was 0–1 in 68.8% of patients, ≥ 2 in 28.6%, and unknown in 2.6%.

**Table 1:** Baseline patient and disease characteristics in the safety analysis set

Characteristics, *n* (%)	SAS (*n* = 77)
Sex	
Male	47 (61.0)
Female	30 (39.0)
Age, years	
Median (range)	73.0 (51.0–88.0)
< 70	17 (22.1)
≥ 70	60 (77.9)
Hospital status	
Inpatient	35 (45.5)
Outpatient	40 (52.0)
Unknown/not specified	2 (2.6)
History of allergy	
Yes	17 (22.1)
No	57 (74.0)
Unknown/not specified	3 (3.9)
Clinical type at disease onset	
Acute-type	34 (44.2)
Lymphoma-type	31 (40.3)
Chronic-type with poor prognostic factors	8 (10.4)
Others^a^	3 (3.9)
Unknown/not specified	1 (1.3)
Ann Arbor classification at onset	
Stage I	7 (9.1)
Stage II	3 (3.9)
Stage III	18 (23.4)
Stage IV	46 (59.7)
Unknown/not specified	3 (3.9)
ECOG PS	
0	25 (32.5)
1	28 (36.4)
2	8 (10.4)
3	13 (16.9)
4	1 (1.3)
Unknown/not specified	2 (2.6)
Target lesions^b^	
Yes	62 (80.5)
No	9 (11.7)
Unconfirmed	4 (5.2)
Unknown/not specified	2 (2.6)
Past medical history	
Yes	40 (52.0)
No	37 (48.1)
Presence of complications	
Yes	47 (61.0)
No	30 (39.0)
Allo-HSCT history	
Yes	5 (6.5)
No	72 (93.5)
History of ultraviolet therapy	
Yes	2 (2.6)
No	75 (97.4)
Number of prior regimens	
1	29 (37.7)
2	24 (31.2)
3	11 (14.3)
4	12 (15.6)
≥ 5	1 (1.3)
Prior regimen history^c^	
Mogamulizumab	44 (57.1)
CHOP	30 (39.0)
VCAP-AMP-VECP	26 (33.8)
Other	48 (62.3)
Best response to the last prior regimen	
CR or PR	33 (42.9)
SD	16 (20.8)
PD	24 (31.2)
Unknown/not specified	4 (5.2)
PD as best response in any prior regimens	
Yes	31 (40.3)
No	46 (59.7)

^a^Smoldering-type ATLL and chronic-type ATLL without poor prognostic factors

^b^Refer to the Japanese Society of Hematology Practical guidelines for hematological malignancies: Adult T-cell leukemia/lymphoma (2013)[1]

^c^Multiple regimens allowed

*Allo-HSCT* allogenic hematopoietic stem cell transplantation, *CHOP* cyclophosphamide, doxorubicin, vincristine, prednisone, *CR* complete response, *ECOG PS* Eastern Cooperative Oncology Group Performance Status, *PD* progressive disease, *PR* partial response, *SAS* safety analysis set, *SD* stable disease, *VCAP-AMP-VECP* vincristine, cyclophosphamide, doxorubicin and prednisolone, doxorubicin, ranimustine and prednisolone; vindesine, etoposide, carboplatin and prednisolone

Twenty-nine patients (37.7%) had received only one prior regimen, 24 (31.2%) two, 11 (14.3%) three, 12 (15.6%) four, and one (1.3%) five or more prior regimens. Regarding prior regimens, 57.1% of patients had previously received mogamulizumab, 39.0% had received combination cyclophosphamide, doxorubicin, vincristine and prednisone (CHOP) chemotherapy and 33.8% had received vincristine, cyclophosphamide, doxorubicin and prednisolone; doxorubicin, ranimustine and prednisolone; vindesine, etoposide, carboplatin and prednisolone (VCAP-AMP-VECP). Five patients (6.5%) had a history of allo-HSCT.

The most frequently reported best responses to the last prior regimen before lenalidomide initiation were CR/PR (42.9%), followed by PD (31.2%) and SD (20.8%).

### Treatment exposure

In the SAS, the mean ± sd initial lenalidomide daily dose was 14.6 ± 6.6 mg and the mean ± sd dose per administration was 14.1 ± 6.5 mg (Table 2). The mean ± sd total treatment duration (including dose interruptions) was 65.1 ± 62.7 days, and the mean ± sd actual treatment duration (not including dose interruptions) was 49.0 ± 50.2 days. Over the treatment period, the mean ± sd total lenalidomide dose was 688.0 ± 880.8 mg and the median (range) total lenalidomide dose was 322.5 (30–4350) mg.

**Table 2:** Dosing of lenalidomide and use of other antineoplastic drugs to treat relapsed/refractory adult T-cell leukemia/lymphoma

	SAS (*n* = 77)
Initial dose, mg	
Mean ± sd	14.6 ± 6.6
Median (range)	15.0 (5–25)
Average dose per administration, mg	
Mean ± sd	14.1 ± 6.5
Median (range)	12.0 (5–25)
Total dose, mg	
Mean ± sd	688.0 ± 880.8
Median (range)	322.5 (30–4350)
Total treatment duration, days (including interruptions)^a^	
Mean ± sd	65.1 ± 62.7
Median (range)	37.0 (3–187)
Actual treatment duration, days (not including interruptions)^b^	
Mean ± sd	49.0 ± 50.2
Median (range)	27.0 (3–184)

^a^Number of days from date of first to last dose of lenalidomide

^b^Number of days with actual lenalidomide administration

*SAS* safety analysis set, *sd* standard deviation

In both the total and actual treatment periods, the majority of patients received lenalidomide for ≤ 90 days (*n* = 53 [68.8%] and *n* = 66 [85.7%], respectively). Overall, 66 patients (85.7%) discontinued lenalidomide treatment (Table 3). The most common reason for discontinuation was primary disease progression (50.7%), followed by AE occurrence whether or not attributable to lenalidomide (41.6%). Among 32 patients with an AE leading to discontinuation, there were 25 AEs (32.5%) considered to be due to lenalidomide, of which the most frequent were rash (9.1%) and erythema multiforme (3.9%). One patient discontinued due to unknown reasons (Table 3).

**Table 3:** Reasons for treatment discontinuation in the safety analysis set

*n* (%)	SAS (*n* = 77)
Patients continuing in the PMS	11 (14.3)
Patients who discontinued the PMS^a^	66 (85.7)
Reasons for discontinuation	
Primary disease progression	39 (50.7)
AEs^b^	32 (41.6)
Death^c^	2 (2.6)
Transfer to another hospital (not due to AEs)	2 (2.6)
Others^d^	2 (2.6)
Patient preference (not due to AEs)	1 (1.3)
Unknown/unstated	1 (1.3)

^a^The reasons for discontinuation were tabulated in duplicate (i.e., multiple reasons for discontinuation could be selected by each patient)

^b^Whether or not attributable to lenalidomide; among 32 cases due to AE, 25 cases (32.5%) were judged to be due to lenalidomide-related ADRs, of which the most frequent were rash (9.1%), erythema multiforme (3.9%), and 2.6% each of erythema, neutrophil count decreased, and platelet count decreased

^c^Due to disease progression (*n* = 2)

^d^General physical health deterioration with no clear or dominant signs or symptoms (*n* = 2)

*AE* adverse event, *PMS* post-marketing surveillance, *SAS* safety analysis set

### Safety

At a median (range) follow-up of 65 (31–187) days, 49 patients (63.6%) in the SAS experienced an ADR; Grade ≥ 3 ADRs occurred in 33 patients (42.9%; Table 4). By SOC, the most common ADRs of any grade (occurring in ≥ 10% of patients) were skin and subcutaneous tissue disorders (39.0%), followed by laboratory abnormalities (18.2%), general disorders and administration site conditions (15.6%), and blood and lymphatic system disorders (10.4%). The most common ADRs by PT of any grade (occurring in ≥ 5.0% of patients) were rash (14.3%), platelet count decreased/thrombocytopenia (14.3%), neutrophil count decreased/neutropenia (13.0%), malaise (7.8%), pyrexia (7.8%), and anemia or erythema multiforme (5.2% each).

**Table 4:** Adverse drug reactions occurring in at least 20% of patients or of Grade ≥ 3 in the safety analysis set

ADRs by SOC ADRs by PT	*n* (%)	
	Any grade	Grade ≥ 3
Total	49 (63.6)	33 (42.9)
Infections and infestations	7 (9.1)	3 (3.9)^a^
Infection	1 (1.3)	1 (1.3)
Staphylococcal sepsis	1 (1.3)	1 (1.3)
Bacterial pyelonephritis	1 (1.3)	1 (1.3)
Pneumonia bacterial	1 (1.3)	1 (1.3)
Neoplasms benign, malignant, and unspecified (including cysts and polyps)	1 (1.3)	1 (1.3)^b^
ATLL	1 (1.3)	1 (1.3)^b^
Blood and lymphatic system disorders	8 (10.4)	6 (7.8)^a^
Anemia	4 (5.2)	3 (3.9)
Myelosuppression	2 (2.6)	1 (1.3)^a^
Febrile neutropenia	1 (1.3)	1 (1.3)
Neutropenia	1 (1.3)	1 (1.3)
Thrombocytopenia	1 (1.3)	1 (1.3)
Metabolism and nutrition disorders	3 (3.9)	2 (2.6)
Decreased appetite	2 (2.6)	1 (1.3)
Hyperkalemia	1 (1.3)	1 (1.3)
Nervous system disorders	7 (9.1)	1 (1.3)
Cognitive disorder	1 (1.3)	1 (1.3)
Hepatobiliary disorders	2 (2.6)	1 (1.3)
Hepatic function abnormal	2 (2.6)	1 (1.3)
Skin and subcutaneous tissue disorders	30 (39.0)	14 (18.2)^a^
Rash	11 (14.3)	4 (5.2)
Erythema multiforme	4 (5.2)	3 (3.9)
Dermatitis exfoliative generalized	3 (3.9)	2 (2.6)
Rash popular	3 (3.9)	2 (2.6)
Erythema	2 (2.6)	1 (1.3)
Drug eruption	2 (2.6)	1 (1.3)
Pruritus	2 (2.6)	1 (1.3)^a^
Rash maculo-papular	2 (2.6)	1 (1.3)
Toxic epidermal necrolysis	1 (1.3)	1 (1.3)
Renal and urinary disorders	1 (1.3)	1 (1.3)
Acute kidney injury	1 (1.3)	1 (1.3)
General disorders and administration site conditions	12 (15.6)	2 (2.6)^a^
Malaise	6 (7.8)	2 (2.6)^a^
Laboratory abnormalities	14 (18.2)	13 (16.9)^a^
Platelet count decreased	10 (13.0)	8 (10.4)
Neutrophil count decreased	9 (11.7)	8 (10.4)^a^
WBC count decreased	2 (2.6)	2 (2.6)

^a^ADR (*n* = 1) of unknown grade was separately reported

^b^Grade 5 *n* = 1

*ADR* adverse drug reaction, *ATLL* adult T-cell lymphoma/leukemia, *PT* preferred term, *SOC* system organ class, *WBC* white blood cell

The most common Grade ≥ 3 ADRs by SOC were skin and subcutaneous tissue disorders (18.2%), followed by laboratory abnormalities (16.9%). The most common Grade ≥ 3 ADRs by PT (occurring in ≥ 5.0% of patients) were neutrophil count decreased/neutropenia and platelet count decreased/thrombocytopenia (11.7% each), and rash (5.2%).

SADRs occurred in 26 patients (33.8%) in the SAS (Supplementary Table S2). The most common SADR by SOC was skin and subcutaneous tissue disorders (16.9%). By PT, the only SADR occurring in ≥ 5.0% of patients was neutrophil count decreased/neutropenia (6.5%). One patient (1.3%) in the SAS experienced a fatal ADR (ATLL disease progression) and one patient (1.3%) with prior mogamulizumab experienced toxic epidermal necrolysis (TEN), which resolved after discontinuation of lenalidomide.

Patients received prophylactic medications, including a combination of trimethoprim and sulfamethoxazole (44.2%), antiviral therapies (16.9%; acyclovir administered to 13.0%) and fluconazole (16.9%).

In the univariate analysis of patient characteristics that may influence the safety of lenalidomide treatment, patients with a history of allergies (*p* = 0.0158), a past medical history (*p* = 0.0311), and the presence of complications (*p* = 0.0031) had significantly higher ADR occurrence (Supplementary Table S3).

### Effectiveness

Among the 65 patients in the EAS, objective response (i.e., CR + PR) was achieved in 19 patients (29.2%), including one with CR (1.5%) and 18 with PR (27.7%; Table 5). The median (range) time to response in the EAS was 59.0 (18–179) days.

**Table 5:** Best response to lenalidomide in patients with relapsed/refractory adult T-cell leukemia/lymphoma in the effectiveness analysis set stratified by age

Best overall response, *n* (%)	EAS *n* = 65	< 70 years *n* = 15	≥ 70 years *n* = 50
Objective response (CR + PR)	19 (29.2)	5 (33.3)	14 (28.0)
CR	1 (1.5)	0	1 (2.0)
PR	18 (27.7)	5 (33.3)	13 (26.0)
SD	12 (18.5)	2 (13.3)	10 (20.0)
PD	25 (38.5)	7 (46.7)	18 (36.0)
Unable to assess	6 (9.2)	1 (6.7)	5 (10.0)
Not evaluated^a^	3 (4.6)	0	3 (6.0)

^a^Responses that were not assessed within the observation period were treated as “not evaluated”

*CR* complete response, *EAS* effectiveness analysis set, *PD* progressive disease, *PR* partial response, *SD* stable disease

*Reference*: Japanese Society of Hematology. Practical guidelines for hematological malignancies: Adult T-cell leukemia/lymphoma [in Japanese]. 2013. http://www.jshem.or.jp/gui-hemali2013/2_8.html#soron. Accessed September 4 2023

**Fig. 2:**
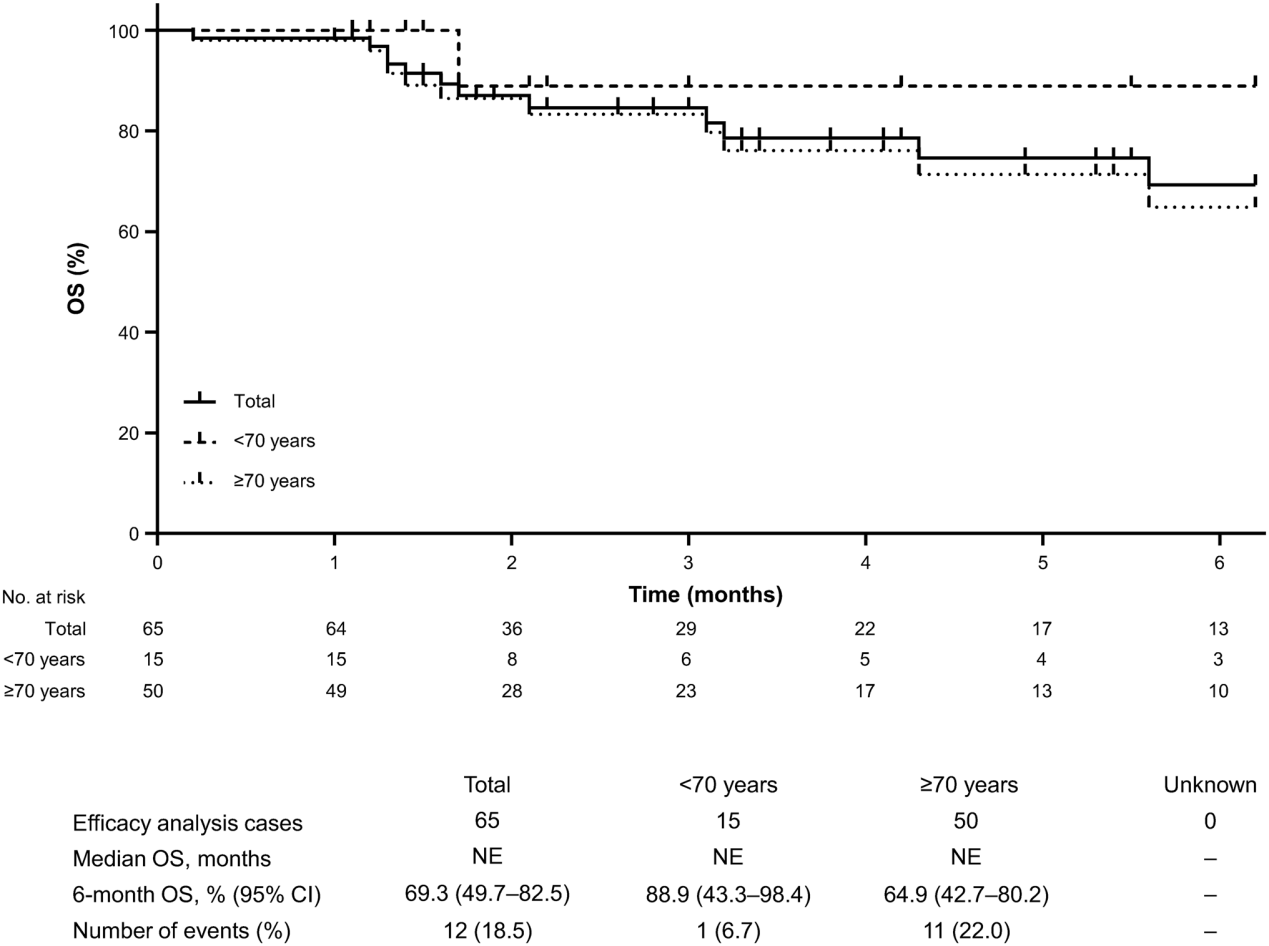
Six-month overall survival of patients with relapsed/refractory adult T-cell leukemia/lymphoma treated with lenalidomide. *CI* confidence interval, *NE* not evaluable, *No*. number, *OS* overall survival

When treatment response was assessed by age (< 70 years [*n* = 15] vs ≥ 70 years [*n* = 50]), no statistically significant differences in the objective response rates (ORR) were observed (33.3% vs 28.0%, respectively; *p* = 0.6904). The 6-month OS rate (95% CI) was 69.3% (49.7–82.5; Fig. 2). When assessed by age, the 6-month OS rate was numerically higher in patients aged < 70 years than in those aged ≥ 70 years (88.9% vs 64.9%, respectively).

In the univariate analysis of background factors that may influence the effectiveness of lenalidomide treatment, patients who were receiving outpatient treatment (*p* = 0.0026), had a history of ultraviolet therapy (*p* = 0.0254) had significantly higher CR + PR response rates (Supplementary Table S4). Additionally, a statistically significant difference in ORR was observed among ECOG PS scores (*p* = 0.0231).

## Discussion

This prospective PMS confirmed the real-world safety of lenalidomide (as assessed by ADR incidence) and clinical effectiveness in Japanese patients with R/R ATLL, in line with results from the ATLL-002 trial [17].

While 41.6% of patients in the SAS discontinued treatment due to an AE, whether or not attributable to lenalidomide, no new safety signals of note were observed. Other real-world data on lenalidomide has documented neutropenia and thrombocytopenia as common ADRs [22], as with this current PMS, in which the most common Grade ≥ 3 ADRs by PT were neutrophil count decreased/neutropenia and platelet count decreased/thrombocytopenia (11.7% each). These findings are consistent with earlier phase 1 trials of lenalidomide, in which neutropenia and thrombocytopenia were also common Grade ≥ 3 AEs (62.0% and 31.0%, respectively) [23]. Similarly, neutropenia and thrombocytopenia (65.4% and 23.1%, respectively) were also frequent Grade ≥ 3 AEs in the ATLL-002 trial [17].

In the current PMS, the incidences of neutropenia (any grade or Grade ≥ 3: 1.3%) and thrombocytopenia (any grade or Grade ≥ 3: 1.3%) were considerably lower than those reported in the ATTL-002 trial for neutropenia (any grade: 73.1%; Grade ≥ 3: 65.4%) and thrombocytopenia (any grade: 76.9%; Grade ≥ 3: 23.1%) [17]. This may be partly explained by differences in the initial dose; in this PMS, the initial dose could be reduced at the discretion of the physician, whereas in ATLL-002, the dose was uniformly started at 25 mg. According to the univariate analysis in the current PMS, patients with comorbidities and a history of allergies, tended to have higher incidences of ADRs, suggesting that these subpopulations should be carefully monitored. In particular, the presence of renal disease as a comorbidity may have been more relevant to the incidence of ADRs (100.0% vs 61.1%; *p* = 0.0805).

The current PMS included five patients with a history of allo-HSCT, of whom two responded to lenalidomide treatment. Also in this PMS, one patient with a history of allo-HSCT and mogamulizumab experienced acute GvHD (Grade 2) during lenalidomide administration, which was manageable by dose modification of lenalidomide. Of note, physicians should carefully monitor for GvHD. Previous retrospective studies have reported newly developing or worsening of GvHD symptoms in patients with ATLL who had undergone allo-HSCT prior to receiving lenalidomide [22, 24]. All of these findings suggest that lenalidomide could be a viable treatment option in patients with ATLL, although patients with prior allo-HSCT should be carefully monitored for GvHD.

The current PMS reported an ORR (i.e., CR + PR) of 29.2% and a 6-month OS rate of 69.3% with lenalidomide. In the ATLL-002 trial, the ORR (i.e., CR + unconfirmed CR + PR) and 6-month OS rates were 42.0% and 100%, respectively [17]. Variations in response rates may be partly attributed to differences in patient characteristics. For instance, in this PMS, 31.2% of patients had PD as the best response with their most recent regimen prior to lenalidomide; however, in ATLL-002, patients were only enrolled if they achieved SD or better with their most recent treatment regimen [17]. Additionally, in the current PMS, 18.2% of all patients had an ECOG PS of 3–4, whereas the ATLL-002 study excluded such patients. Therefore, the poorer survival outcomes observed in the current PMS are likely to be directly influenced by the patients’ disease status at baseline. Patients who had PD as their best response to their most recent prior regimen had an ORR of 22.2% in the current PMS, thereby confirming the effectiveness of lenalidomide in refractory ATLL patients who were excluded from ATLL-002 [17]. The current PMS enrolled older patients (median age: 73 years) compared with the ATLL-002 trial (median age: 68.5 years); although, based on the univariate analysis, no statistically significant differences in response rates were observed between patients aged < 70 versus ≥ 70 years (33.3% vs 28.0%, respectively, *p* = 0.6904).

The antibody mogamulizumab is also approved for ATLL in Japan [25]. In our PMS, the ORR in patients who had previously received mogamulizumab (*n* = 38) was 31.6%, similar to the ORR in the overall population. Additionally, the incidence of any grade ADRs in patients previously treated with mogamulizumab was comparable with that observed in the overall SAS (72.7% vs 63.6%, respectively). Moreover, the incidence of Grade ≥ 3 ADRs was comparable in each prior regimen group (mogamulizumab: 52.3%, CHOP: 50.0%, VCAP-AMP-VECP: 50.0%). These findings indicate that lenalidomide may be a safe and effective treatment option for patients with R/R ATLL who have previously received mogamulizumab.

More recently, tucidinostat (a histone deacetylase inhibitor) and valemetostat (an enhancer of zeste 1 polycomb repressive complex 2 subunit inhibitor) have also been approved for R/R ATLL in Japan [26, 27]. A phase 2b trial of 23 R/R ATLL patients receiving tucidinostat reported an ORR and median OS of 30.4% and 7.9 months, respectively [28]. Another phase 2 trial of 25 R/R ATLL patients treated with valemetostat reported a clinical response rate of 48.0% and a median OS of 16.4 months [29]. Given that the clinical trials of these new agents have also included patients with refractory disease, these new agents have shown some improvement in efficacy compared with that reported in the ATLL-002 trial to date [17]. However, there are still several unmet medical needs for patients with R/R ATLL.

This PMS has some limitations, mainly relating to the nature of its design (e.g., open-label and uncontrolled) which may have introduced several biases (e.g., selection, attrition, and information bias). Additionally, as the outcomes were not centrally reviewed, the reliability of these results were dependent on the accuracy of the physician’s assessment.

In conclusion, no new safety signals of lenalidomide have been observed in this PMS compared with the ATLL-002 trial. Lenalidomide also displayed a favorable benefit-risk balance in Japanese patients with R/R ATLL treated in routine clinical practice.

## Supplementary Information

Below is the link to the electronic supplementary material.

Supplementary file1 (DOCX 62 KB)

## Data Availability

Bristol Myers Squibb policy on data sharing may be found at https://www.bms.com/researchers-and-partners/clinical-trials-and-research/disclosure-commitment.html.
