# Supplementary material for: Safety and effectiveness of lenalidomide in Japanese patients with relapsed/refractory ATLL: post-marketing surveillance
Source: Int J Hematol. 2024 Nov 21;121(1):79–88. doi: 10.1007/s12185-024-03864-3 (PMC11742342; doi:10.1007/s12185-024-03864-3)
Supplement: Supplementary file 1 — Supplementary file1 (DOCX 62 KB) [file 12185_2024_3864_MOESM1_ESM.docx]

**Safety and effectiveness of lenalidomide in Japanese patients with relapsed/refractory ATLL: post-marketing surveillance**

**Authors:** Tohru Miyazaki^1^, Shuji Uno^1^, Hiroaki Fujimori^1^ and Yoko Motegi^2^

**Affiliations:** ^1^Japan Medical-Hematology, Bristol Myers Squibb, Tokyo, Japan; ^2^PMS Operations, Worldwide Patient Safety Japan, Bristol Myers Squibb, Tokyo, Japan.

**Corresponding author:** Tohru Miyazaki

Japan Medical-Hematology, Bristol Myers Squibb, Otemachi One Tower, 1-2-1 Otemachi, Chiyoda-ku, Tokyo, 100-0004, Japan

**Tel:** +81-80-8146-9526; **Email:** Tohru.Miyazaki@bms.com

# Supplementary material

## Supplementary Table S1.

Responses according to the JCOG version of the Criteria for Determining Treatment Efficacy for ATLL [1]

| **Treatment response^a^** | **Target lesions** | | **Non-target lesions** | | **Overall** | | | |
| --- | --- | --- | --- | --- | --- | --- | --- | --- |
|  | **Nodal** | **Extranodal** | **Nodal** | **Extranodal** | **Skin lesions** | **Peripheral blood lesions** | **Bone marrow infiltration** | **New lesions** |
| **Target lesions present at baseline** | | | | | | | | |
| CR | Normal | Absent | Normal | Absent | Normal | Normal | Negative | No |
| PR | ≥50% decrease in SPD | ≥50% decrease in SPD | Normal or no increase | Absent or no increase | Normal or decreased | Normal or decreased | – | No |
| SD | Not CR, PR or PD | | | | | | | |
| PD^b^ | ≥50% SPD or (re) enlargement | ≥50% SPD or (re) appearance | (Re) enlargement | (Re) appearance | Increase or reappearance | Increase | Positive | Yes |
| **Target lesions absent at baseline** | | | | | | | | |
| CR | – | – | Normal | Absent | Normal | Normal | Negative | No |
| PR | – | – | Normal or no increase | Absent or no increase | Normal or decreased | Normal or decreased | – | No |
| SD | Not CR, PR or PD | | | | | | | |
| PD^b^ | – | – | (Re) enlargement | (Re) appearance | Increase or reappearance | Increase | Positive | Yes |

^a^If any of the below criteria were unevaluable, the overall rating should be ‘Unevaluable’

^b^Patients who meet at least one of the criteria are assesses as having PD

ATLL, adult T-cell leukemia/lymphoma; CR, complete response; JCOG, Japanese Clinical Oncology Group; PD, progressive disease; PR, partial response SPD, sum of the products of the greatest diameter

## Supplementary Table S2.

Serious adverse drug reactions

| **SOC**  **PT** | **n (%)** |
| --- | --- |
| Total | 26 (33.8) |
| Infections and infestations | 4 (5.2)^a^ |
| Infection | 1 (1.3) |
| Pneumonia | 1 (1.3) |
| Staphylococcal sepsis | 1 (1.3) |
| Bacterial pyelonephritis | 1 (1.3) |
| Pneumonia bacteria | 1 (1.3) |
| Neoplasms benign, malignant and unspecified (including cysts and polyps) | 1 (1.3) |
| ATLL | 1 (1.3) |
| Blood and lymphatic system disorders | 2 (2.6)^a^ |
| Myelosuppression | 1 (1.3)^a^ |
| Neutropenia | 1 (1.3) |
| Immune system disorders | 1 (1.3) |
| Acute graft versus host disease in skin | 1 (1.3) |
| Metabolism and nutrition disorders | 2 (2.6) |
| Hyperkalemia | 1 (1.3) |
| Decreased appetite | 1 (1.3) |
| Nervous system disorders | 1 (1.3) |
| Cognitive disorder | 1 (1.3) |
| Eye disorders | 1 (1.3) |
| Lacrimation increased | 1 (1.3) |
| Respiratory, thoracic and mediastinal disorders | 1 (1.3) |
| Wheezing | 1 (1.3) |
| Hepatobiliary disorders | 1 (1.3) |
| Hepatic function abnormal | 1 (1.3) |
| Skin and subcutaneous tissue disorders | 13 (16.9) |
| Erythema multiforme | 3 (3.9) |
| Rash | 3 (3.9) |
| Dermatitis exfoliative generalized | 2 (2.6) |
| Drug eruption | 1 (1.3) |
| Erythema | 1 (1.3) |
| Pruritus | 1 (1.3) |
| Rash maculo-papular | 1 (1.3) |
| Rash papular | 1 (1.3) |
| Toxic epidermal necrolysis | 1 (1.3) |
| Renal and urinary disorders | 1 (1.3) |
| Acute kidney injury | 1 (1.3) |
| General disorders and administration site conditions | 2 (2.6) |
| Malaise | 1 (1.3) |
| Pyrexia | 1 (1.3) |
| Laboratory abnormalities | 6 (7.8)^a^ |
| Neutrophil count decreased | 4 (5.2)^a^ |
| Platelet count decreased | 3 (3.9) |
| WBC count decreased | 1 (1.3) |

^a^ADR (n=1) of unknown grade was separately reported

ATLL, adult T-cell leukemia/lymphoma; PT, preferred term; SOC, system organ class; WBC, white blood cell

## Supplementary Table S3.

Factors affecting safety (occurrence of adverse drug reactions) of lenalidomide in relapsed/refractory adult T-cell leukemia/lymphoma in the safety analysis set

| **Background factor** | **Category** | **Total, n** | **ADRs, n (%)** | **Chi-square test** | **Cochran-Armitage test** |
| --- | --- | --- | --- | --- | --- |
| Total | – | 77 | 49 (63.6) | – | – |
| Sex | Male | 47 | 30 (63.8) | p=0.9648 | – |
|  | Female | 30 | 19 (63.3) |  |  |
| Age | <70 years | 17 | 9 (52.9) | p=0.2990 | – |
|  | ≥70 years | 60 | 40 (66.7) |  |  |
| Hospital status | Inpatient | 35 | 23 (65.7) | p=0.6098 | – |
|  | Outpatient | 40 | 24 (60.0) |  |  |
|  | Unknown/not specified | 2 | 2 (100.0) |  |  |
| History of allergy | Yes | 17 | 15 (88.2) | p=0.0158 | – |
|  | No | 57 | 32 (56.1) |  |  |
|  | Unknown/not specified | 3 | 2 (66.7) |  |  |
| Clinical type at disease onset | Acute-type | 34 | 21 (61.8) | p=0.9951 | – |
|  | Lymphoma-type | 31 | 20 (64.5) |  |  |
|  | Chronic-type with poor prognostic factors | 8 | 5 (62.5) |  |  |
|  | Others | 3 | 2 (66.7) |  |  |
|  | Unknown/not specified | 1 | 1 (100.0) |  |  |
| Ann Arbor classification at onset | Stage I | 7 | 4 (57.1) | p=0.5221 | p=0.9945 |
|  | Stage II | 3 | 3 (100.0) |  |  |
|  | Stage III | 18 | 10 (55.6) |  |  |
|  | Stage IV | 46 | 29 (63.0) |  |  |
|  | Unknown/not specified | 3 | 3 (100.0) |  |  |
| ECOG PS | 0 | 25 | 15 (60.0) | p=0.3360 | p=0.4482 |
|  | 1 | 28 | 21 (75.0) |  |  |
|  | 2 | 8 | 4 (50.0) |  |  |
|  | 3 | 13 | 6 (46.2) |  |  |
|  | 4 | 1 | 1 (100.0) |  |  |
|  | Unknown/not specified | 2 | 2 (100.0) |  |  |
| Target lesion | Yes | 62 | 39 (62.9) | p=0.1915 | – |
|  | No | 9 | 7 (77.8) |  |  |
|  | Unconfirmed | 4 | 1 (25.0) |  |  |
|  | Unknown/not specified | 2 | 2 (100.0) |  |  |
| Past medical history | Yes | 40 | 30 (75.0) | p=0.0311 | – |
|  | No | 37 | 19 (51.4) |  |  |
| Prescence of comorbidities | Yes | 47 | 36 (76.6) | p=0.0031 | – |
|  | No | 30 | 13 (43.3) |  |  |
| Allo-HSCT history | Yes | 5 | 4 (80.0) | p=0.4315 | – |
|  | No | 72 | 45 (62.5) |  |  |
| History of ultraviolet therapy | Yes | 2 | 2 (100.0) | p=0.2787 | – |
|  | No | 75 | 47 (62.7) |  |  |
| Number of prior regimens | 1 | 29 | 14 (48.3) | p=0.0669 | p=0.4873 |
|  | 2 | 24 | 20 (83.3) |  |  |
|  | 3 | 11 | 7 (63.6) |  |  |
|  | 4 | 12 | 8 (66.7) |  |  |
|  | ≥5 | 1 | 0 |  |  |
| Initial dose | <10 mg | 10 | 5 (50.0) | p=0.2242 | p=0.4519 |
|  | ≥10 to <15 mg | 23 | 17 (73.9) |  |  |
|  | ≥15 to <20 mg | 24 | 16 (66.7) |  |  |
|  | ≥20 to <25 mg | 3 | 3 (100.0) |  |  |
|  | ≥25 mg | 17 | 8 (47.1) |  |  |
| Average dose per administration | <10 mg | 14 | 11 (78.6) | p=0.0548 | p=0.0111 |
|  | ≥10 to <15 mg | 30 | 21 (70.0) |  |  |
|  | ≥15 to <20 mg | 12 | 6 (50.0) |  |  |
|  | ≥20 to <25 mg | 5 | 4 (80.0) |  |  |
|  | ≥25 mg | 13 | 4 (30.8) |  |  |
|  | Unknown/not specified | 3 | 3 (100.0) |  |  |
| Prior regimen history^a^ | Mogamulizumab | 44 | 32 (72.7) | – | – |
|  | CHOP | 30 | 19 (63.3) |  |  |
|  | VCAP-AMP-VECP | 26 | 18 (69.2) |  |  |
|  | Other | 48 | 30 (62.5) |  |  |
| Best response to the last prior regimen | CR or PR | 33 | 23 (69.7) | p=0.6445 | p=0.7603 |
|  | SD | 16 | 9 (56.3) |  |  |
|  | PD | 24 | 16 (66.7) |  |  |
|  | Unknown/not specified | 4 | 1 (25.0) |  |  |
| PD as best response in any prior regimens | Yes | 31 | 20 (64.5) | p=0.8952 | – |
|  | No | 46 | 29 (63.0) |  |  |

^a^Multiple regimens allowed

ADR, adverse drug reaction; allo-HSCT, allogenic hematopoietic stem cell transplantation; CHOP, cyclophosphamide, doxorubicin, vincristine, prednisone; CR, complete response; ECOG PS, Eastern Cooperative Oncology Group Performance Status; PD, progressive disease; PR, partial response; SD, stable disease; VCAP-AMP-VECP, vincristine, cyclophosphamide, doxorubicin and prednisolone; doxorubicin, ranimustine and prednisolone; vindesine, etoposide, carboplatin and prednisolone

## Supplementary Table S4.

Factors affecting response rates (complete response/partial response) of lenalidomide in relapsed/refractory adult T-cell leukemia/lymphoma in the effectiveness analysis set

| **Background factor** | **Category** | **Total, n** | **CR/PR, n (%)** | **Chi-square test** | **Cochran-Armitage test** |
| --- | --- | --- | --- | --- | --- |
| Total | – | 65 | 19 (29.2) | – | – |
| Sex | Male | 37 | 12 (32.4) | p=0.5141 | – |
|  | Female | 28 | 7 (25.0) |  |  |
| Age | <70 years | 15 | 5 (33.3) | p=0.6904 | – |
|  | ≥70 years | 50 | 14 (28.0) |  |  |
| Hospital status | Inpatient | 28 | 3 (10.7) | p=0.0026 | – |
|  | Outpatient | 35 | 16 (45.7) |  |  |
|  | Unknown/not specified | 2 | 0 |  |  |
| History of allergy | Yes | 14 | 5 (35.7) | p=0.6401 | – |
|  | No | 48 | 14 (29.2) |  |  |
|  | Unknown/not specified | 3 | 0 |  |  |
| Clinical type at disease onset | Acute-type | 29 | 10 (34.5) | p=0.4095 | – |
|  | Lymphoma-type | 26 | 5 (19.2) |  |  |
|  | Chronic-type with poor prognostic factors | 6 | 3 (50.0) |  |  |
|  | Others | 3 | 1 (33.3) |  |  |
|  | Unknown/not specified | 1 | 0 |  |  |
| Ann Arbor classification at onset | Stage I | 5 | 2 (40.0) | p=0.3085 | p=0.5574 |
|  | Stage II | 2 | 0 |  |  |
|  | Stage III | 15 | 2 (13.3) |  |  |
|  | Stage IV | 40 | 14 (35.0) |  |  |
|  | Unknown/not specified | 3 | 1 (33.3) |  |  |
| ECOG PS | 0 | 21 | 9 (42.9) | p=0.1136 | p=0.0231 |
|  | 1 | 26 | 8 (30.8) |  |  |
|  | 2 | 6 | 2 (33.3) |  |  |
|  | 3 | 10 | 0 |  |  |
|  | 4 | 0 | 0 |  |  |
|  | Unknown/not specified | 2 | 0 |  |  |
| Target lesions | Yes | 54 | 16 (29.6) | p=0.4949 | – |
|  | No | 7 | 3 (42.9) |  |  |
|  | Not confirmed | 2 | 0 |  |  |
|  | Unknown/not specified | 2 | 0 |  |  |
| Past medical history | Yes | 35 | 10 (28.6) | p=0.8995 | – |
|  | No | 30 | 9 (30.0) |  |  |
| Presence of comorbidities | Yes | 38 | 9 (23.7) | p=0.2435 | – |
|  | No | 27 | 10 (37.0) |  |  |
| Allo-HSCT history | Yes | 5 | 2 (40.0) | p=0.5816 | – |
|  | No | 60 | 17 (28.3) |  |  |
| History of ultraviolet therapy | Yes | 2 | 2 (100.0) | p=0.0254 | – |
|  | No | 63 | 17 (27.0) |  |  |
| Number of prior regimens | 1 | 25 | 7 (28.0) | p=0.8997 | p=0.9659 |
|  | 2 | 21 | 6 (28.6) |  |  |
|  | 3 | 10 | 4 (40.0) |  |  |
|  | 4 | 8 | 2 (25.0) |  |  |
|  | ≥5 | 1 | 0 |  |  |
| Initial dose | <10 mg | 10 | 2 (20.0) | p=0.8064 | p=0.9399 |
|  | ≥10 to <15 mg | 19 | 6 (31.6) |  |  |
|  | ≥15 to <20 mg | 20 | 7 (35.0) |  |  |
|  | ≥20 to <25 mg | 2 | 1 (50.0) |  |  |
|  | ≥25 mg | 14 | 3 (21.4) |  |  |
| Average dose per administration | <10 mg | 13 | 5 (38.5) | p=0.1904 | p=0.1089 |
|  | ≥10 to <15 mg | 25 | 10 (40.0) |  |  |
|  | ≥15 to <20 mg | 10 | 1 (10.0) |  |  |
|  | ≥20 to <25 mg | 4 | 2 (50.0) |  |  |
|  | ≥25 mg | 10 | 1 (10.0) |  |  |
|  | Unknown/not specified | 3 | 0 |  |  |
| Prior regimen history^a^ | Mogamulizumab | 38 | 12 (31.6) | – | – |
|  | CHOP | 28 | 10 (35.7) |  |  |
|  | VCAP-AMP-VECP | 23 | 7 (30.4) |  |  |
|  | Other | 37 | 8 (21.6) |  |  |
| Best response to the last prior regimen | CR/PR | 29 | 11 (37.9) | p=0.5132 | p=0.2501 |
|  | SD | 14 | 4 (28.6) |  |  |
|  | PD | 18 | 4 (22.2) |  |  |
|  | Unknown/not specified | 4 | 0 |  |  |
| PD as best response in any prior regimens | Yes | 23 | 5 (21.7) | p=0.3257 | – |
|  | No | 42 | 14 (33.3) |  |  |

^a^Multiple regimens allowed

Allo-HSCT, allogenic hematopoietic stem cell transplantation; CHOP, cyclophosphamide, doxorubicin, vincristine, prednisone; CR, complete response; ECOG PS, Eastern Cooperative Oncology Group Performance Status; PD, progressive disease; PR, partial response; SD, stable disease; VCAP-AMP-VECP, vincristine, cyclophosphamide, doxorubicin and prednisolone; doxorubicin, ranimustine and prednisolone; vindesine, etoposide, carboplatin and prednisolone

**Reference**

1. Japanese Society of Hematology. Practical guidelines for hematological malignancies: Adult T-cell leukemia/lymphoma [in Japanese]. 2013. <http://www.jshem.or.jp/gui-hemali2013/2_8.html#soron>. Accessed September 4 2023.
